# Oncogene-Induced Senescence as a New Mechanism of Disease: The Paradigm of Erdheim–Chester Disease

**DOI:** 10.3389/fimmu.2014.00281

**Published:** 2014-06-13

**Authors:** Giulio Cavalli, Riccardo Biavasco, Bruno Borgiani, Lorenzo Dagna

**Affiliations:** ^1^Unit of Internal Medicine and Clinical Immunology, IRCCS San Raffaele Scientific Institute, Milan, Italy; ^2^Vita-Salute San Raffaele University, Milan, Italy

**Keywords:** Erdheim–Chester disease, histiocytosis, oncogene-induced senescence, BRAF kinases, inflammation, macrophages

## Abstract

Erdheim–Chester disease (ECD) is a rare form of systemic histiocytosis characterized by the diffuse infiltration of tissues by lipid-laden macrophages. As the clinical course and prognosis are highly influenced by site of disease involvement, ECD course ranges from asymptomatic to life threatening, with a reported global 5-year mortality of 30–40%. Whether ECD is an inflammatory or clonal disease in its nature has long been debated. The disease is characterized by a network of pro-inflammatory cyto/chemokines responsible for the recruitment and activation of histiocytes into ECD lesions, similarly to what reported in Langerhans cell histiocytosis (LCH). Growing evidence supports a central role of the oncogenic BRAF^V600E^ mutation in histiocytosis pathogenesis, and suggests oncogene-induced senescence (OIS), a major protective mechanism against oncogenic events characterized by cell-cycle arrest and the induction of pro-inflammatory molecules, as the possible link between the oncogenic mutation and the observed inflammation. Indeed, ECD recapitulates *in vivo* the molecular events associated with OIS, i.e., cell-cycle arrest and a potent local inflammatory response. Accordingly, the infiltration of different tissues by macrophages and the inflammatory local and systemic effects observed in ECD likely represent a drawback of OIS. Therefore, these findings delineate a new conception of OIS as a new pathogenic mechanism intrinsically responsible for disease development.

## Introduction

**Figure 1:**
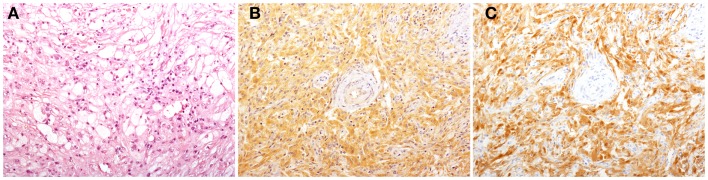
**Histological findings in patients with Erdheim–Chester disease (ECD)**. Histology shows a xanthogranulomatous infiltrate composed by foamy histiocytes accompanied by fibrosis [**(A)**, H&E, original magnification 200×]. Immunohistochemical studies reveal that some of the infiltrating histiocytes stain for BRAF^V600E^ [**(B)**, VE1 immunostaining, 200×], and p16^Ink4a^ [**(C)**, p16^Ink4a^ immunostaining, 200×].

Erdheim–Chester disease (ECD) is a rare, multi-systemic, non-Langerhans form of histiocytosis characterized by the infiltration of different tissues by foamy, lipid-laden macrophages. William Chester first reported the disease in 1930 together with his mentor Jakob Erdheim, a Viennese pathologist (1). The clinical spectrum of ECD is broad, as pathologic histiocytes can infiltrate virtually every organ and tissue (Figure 1). The protean clinical manifestations of ECD include bone pain due to skeletal involvement, diabetes insipidus, neurological and constitutional symptoms, retroperitoneal infiltration with possible ureteral obstruction, as well as pulmonary, cutaneous, cardiovascular, and endocrine involvement (2–4). Although ECD is undoubtedly rare, it is arguably an overlooked diagnosis (5). In recent times, the number of recognized cases increased dramatically due to the raising awareness of ECD in the medical community. Over time, several different therapeutic approaches have been explored, often with unsatisfactory results, and the prognosis has been traditionally considered poor. More recently, it was demonstrated that pathologic histiocytes bear an activating mutation in the oncogene BRAF (BRAF^V600E^) (6–10). This recent discovery led to novel, targeted therapeutic strategies for patients affected by this neglected disease (11).

## Pathogenetic Theories: From Neoplasia to Inflammation

Erdheim–Chester disease is characterized by xanthomatous or xanthogranulomatous infiltration of tissues by foamy histiocytes, or lipid-laden macrophages, surrounded by fibrosis. The pathological cells express markers of the macrophage lineage, such as CD14 and CD68, and in the majority of cases (80%) stain negative for markers of dendritic lineage, such as CD1a and S-100 (2). Such histological/immunohistochemical features help distinguish ECD from Langerhans’ cell histiocytosis (LCH), a disease showing many similarities with ECD (12).

Several different theories on the pathogenesis of ECD arose over time. Based on the aforementioned histopathological findings, ECD was first hypothesized to be a lipid storage disorder, but investigations aimed at confirming this theory were inconclusive (13). Afterward, the conception of ECD as a neoplastic disease became the prevalent theory. This hypothesis was mostly supported by the clinical observation that aberrant macrophages progressively infiltrate different tissues, thus determining an aggressive, multi-systemic clinical course.

Yet, the hypothesis of a neoplastic pathogenesis never found ultimate confirmation, due to the inability to unambiguously identify evidence of clonal proliferation and of impaired cellular differentiation (14–17).

In the following years, most studies aimed at identifying alternative mechanisms possibly responsible for the recruitment, accumulation, and differentiation of histiocytes into affected tissues. Analogously to the “cytokine storm” described in LCH, our group and others discovered in ECD lesions a pro-inflammatory milieu responsible for the skewing of histiocytes toward an M1, inflammatory phenotype (18). Local pro-inflammatory effects were paralleled by the systemic release of a network of Th1-associated soluble factors, such as IL-1, IL-6, CCL2, CCL5, CXCL8, TNF-α, and interferon (IFN)-γ (17, 19–21).

The clinical and pathogenic similarities between ECD and LCH resulted in the adoption of therapeutic strategies for the treatment of ECD that were mostly derived from the clinical experience with LCH. For instance, early reports documented the efficacy of the purine analog cladribine, which has been used in the treatment of multisystem LCH (22, 23). IFN-α, also used in the management of ECD based on its clinical efficacy against LCH, later became the first-line drug for the treatment of ECD, and its efficacy has now been extensively documented (24, 25). The molecular mechanisms underlying the efficacy of IFN-α in ECD are unclear. Among the proposed, disparate biological effects of this drug are the modulation of maturation and activation of dendritic cells, the immune-mediated destruction of histiocytes via natural killer cells, and direct anti-proliferative effects (26–29).

Following the identification of this network of pro-inflammatory mediators in lesions and sera of ECD patients, cytokine blockade with biological drugs was explored as a possible therapeutic strategy. Clinical observations on small numbers of patients demonstrated that treatment with cytokine-blocking agents could be effective in the management of the disease. In particular, treatment with the IL-1 receptor antagonist anakinra was associated to a favorable clinical response in a significant number of ECD patients with different disease manifestations (30–32). More limited clinical experience with TNF-α-blocking drugs also yielded encouraging results (33). Moreover, the results of a phase II clinical trial conducted by our group aimed at evaluating the IL-6 blocker tocilizumab are expected in the near future (NCT01727206). The promising results of cytokine inhibitors in the management of the disease long provided a proof-of-concept to the central pathogenic role of cytokine/chemokine-mediated histiocyte recruitment and activation in the development of ECD lesions.

More recently, new insights into the pathogenesis of ECD came from the discovery that a significant proportion of ECD and LCH patients bear a mutation in the proto-oncogene *BRAF* (7, 10, 34), further substantiating a correspondence between the two diseases. The first identification of a *BRAF* mutation in ECD macrophages was reported by Blombery et al. (10): their investigation revealed the presence of a histiocyte-restricted mutation in the genetic sequence of *BRAF*, which confers the amino acid substitution of glutamic acid for valine at position 600 of the B-Raf protein (BRAF^V600E^). Haroche et al. (7) expanded this finding by examining tissue samples from 127 patients affected by different histiocytoses. The investigation, performed by pyrosequencing and confirmed by immunohistochemical analysis, revealed the presence of BRAF^V600E^ in 13 out of 24 (54%) ECD patients and in 11 out of 29 (38%) LCH patients. The mutation was not found in any patient affected by different histiocytoses. Other studies performed on larger cohorts revealed that 57% (35 out of 61) of LCH patients and 51% (19 out of 37) of ECD patients harbor the BRAF^V600E^ mutation (9, 34). The recent finding of an oncogenic *NRAS* mutation in a BRAF^V600E^-negative ECD patient further supports the hypothesis of significant role of the Ras–Raf–Mek–Erk pathway in the pathogenesis of the disease (35).

The discovery that histiocytes from a considerable proportion of ECD patients bear the BRAF^V600E^ mutation inspired a new therapeutic strategy. The small molecule vemurafenib (also known as PLX4032 and marketed as Zelboraf), was the first specific BRAF^V600E^ inhibitor to be approved by FDA for the treatment of malignant melanomas as well as other cancers (36). By inhibiting the mutated kinase activity, vemurafenib abrogates signaling downstream B-Raf, thus blocking the proliferation and inducing death of cells carrying this mutation (37, 38). When administered to a small number of patients with severe ECD who harbored the mutation, vemurafenib showed dramatic efficacy (11). The clinical efficacy of selective BRAF^V600E^ inhibition demonstrates the crucial relevance of the oncogenic BRAF^V600E^ mutation in the pathogenesis of ECD. Meanwhile, it reinvigorates the hypothesis that ECD might be a clonal disease.

## The BRAF^V600E^ Mutation is Invariably Associated with ECD

In spite of most recent advances in the understanding of ECD pathogenesis, some areas of uncertainty remained unexplored. For instance, BRAF^V600E^ was detectable in a relevant fraction, but not in all ECD patients. Furthermore, the presence of an oncogenic mutation *per se* did not explain the robust local and systemic inflammatory response observed in ECD.

At immunohistochemical analyses, ECD lesions are characterized by an uneven distribution of different cellular populations. Moreover, when we analyzed available specimens from a large cohort of ECD patients followed-up at our Institution, we observed that some samples stained positive for BRAF^V600E^ as evaluated by means of immunohistochemistry, whereas the analysis of the same samples by means of pyrosequencing failed to detect the BRAF^V600E^ mutation (6). BRAF^V600E^ is exclusively found in the histiocytic compartment, but the percentage of BRAF^V600E^-positive histiocytes varies considerably among different biopsy samples, ranging from 20 to 50% (6, 7, 10). On these grounds, we hypothesized that even the ECD biopsy samples that were negative for BRAF^V600E^ as evaluated by pyrosequencing might include a small fraction of BRAF^V600E^-mutated macrophages, which remained undetected due to the higher frequency of the wild-type allele. Indeed, traditional pyrosequencing techniques can lead to the generation of false negatives, since mutated histiocytes may be undetectable when present in very small numbers – e.g., <10% of total cells – due to the overwhelming signal of wild-type cells (6).

We thus re-evaluated the ECD biopsy samples exploiting an ultrasensitive approach, characterized by the amplification of the extracted DNA by means of an *ad hoc* locked nucleic acid (LNA)–PCR/pyrosequencing assay. This combination of techniques – a wild-type allele-specific locked PCR followed by pyrosequencing, further on referred to as LNA/pyrosequencing – enabled the identification of one mutated *BRAF* allele among 10,000 wild-type copies (6, 39). By means of LNA/pyrosequencing, we demonstrated the presence of BRAF^V600E^ in histological samples from 18 out of 18 studied ECD patients, whereas direct pyrosequencing allowed the detection of the mutation in only 12 out of 18 patients (6). Given the extremely high sensitivity of this technique, we also investigated the *BRAF* status in peripheral blood mononuclear cells, and identified the presence of BRAF^V600E^ in a small fraction of cells, which were characterized as circulating monocytes by means of immunohistochemistry and flow cytometry. These data were independently confirmed by droplet-digital PCR. Although the mere association of BRAF^V600E^ with ECD does not imply pathogenic causality, the extremely high occurrence of BRAF^V600E^ in ECD patients suggests that this mutation plays a crucial role in the pathogenesis of the disease. Again, the hypothesis of a pivotal pathogenic role of BRAF^V600E^ in ECD is substantiated by the clinical experience with specific BRAF^V600E^ pharmacologic inhibitors, whose efficacy in controlling ECD manifestations is becoming progressively evident (11).

## BRAF^V600E^ Mutation and Oncogene-Induced Senescence in ECD

B-Raf is a serine–threonine protein kinase that is implicated in the Ras–Raf–Mek–Erk mitogen-activated protein kinase (MAPK) transduction pathway. This signaling pathway is activated by extracellular growth factors binding to membrane tyrosine kinase receptors, and regulates cell proliferation and survival (40). BRAF^V600E^ is characterized by a conformational change that makes the ATP binding site constantly accessible (41), thus causing the constitutive activation of the aberrant protein. The constitutive activation of B-Raf results in the deregulated phosphorylation of downstream signaling proteins and promotes uncontrolled cellular proliferation. Consistently, BRAF^V600E^ is a mutational hotspot in a variety of human cancers, including melanomas, papillary thyroid cancers, and hairy-cell leukemia (42–44).

In addition to this recognized oncogenic activity, BRAF^V600E^ mutation has also been associated with oncogene-induced senescence (OIS), a recently identified major protective mechanism against oncogenic events (45, 46). In OIS, the isolated activation of an oncogene, in the absence of additional mutations, induces cell-cycle arrest and prevents cell proliferation. In this way, OIS ensures the elimination of early neoplastic cells from the proliferative pool, thus dampening the risk of transformation to overt cancer associated with persistent cellular outgrowth. OIS is associated with distinctive molecular features, such as the expression of p16^Ink4a^, a major tumor suppressor protein, and with potent pro-inflammatory effects via the activation of a pro-inflammatory transcriptome. Indeed, BRAF^V600E^-mutated cells produce a variety of Th1-associated cytokines and chemokines whose autocrine and paracrine effects are crucial for the induction and maintenance of the OIS phenotype (47). In ECD, the local production of chemokines by BRAF^V600E^-mutated cells likely attracts circulating leukocytes to the lesion sites, where the inflammatory milieu sustained by senescence-associated cytokines elicit the pro-inflammatory differentiation of recruited cells. The so formed local inflammatory reaction may hinder the transformation of the mutated cells to overt cancer, while perpetuating the OIS phenotype of cells. Recent studies on murine models of *BRAF*-mutated thyroid cancer demonstrated that BRAF^V600E^ confers the capability to the cells harboring the mutation of potently recruiting macrophages (48). Indeed, BRAF^V600E^ induces an increased expression of the macrophage chemo-attractants CXCL8, CCL2, CCL4, and CCL5 (17, 19). As a consequence, *BRAF*-mutated thyroid cancers are densely infiltrated with tumor-associated macrophages, which may account for up to 50% of the total mass of the tumor (48).

## Oncogene-Induced Senescence: A New Pathogenic Mechanism Responsible for Disease Development?

**Figure 2:**
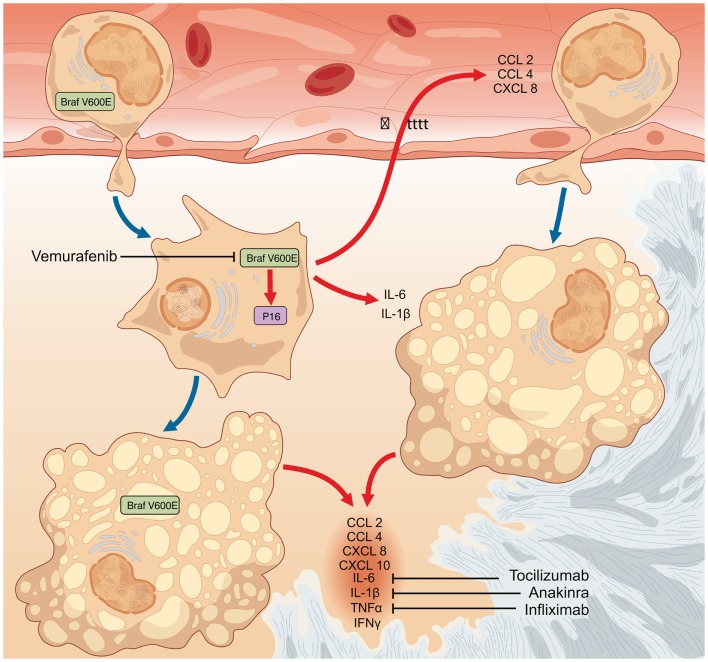
**The finding that a fraction of both circulating monocytes and tissue-infiltrating macrophages bear the BRAF^V600E^ mutation suggests that the oncogenic event likely occurs in a monocyte–macrophage precursor**. The occurrence of the BRAF^V600E^ oncogenic mutation represents the initiating event in the pathogenesis of Erdheim–Chester disease (ECD). In turn, BRAF^V600E^ activates in mutated cells oncogene-induced senescence (OIS) pathways, as also testified by the intense expression of the cell-cycle gatekeeper p16^Ink4a^. OIS results in the activation of a pro-inflammatory transcriptome and in the production of cytokines and chemokines (CCL2, CCL4, CXCL8, CXCL10, IL-1β, IL-6, TNFα, and IFNγ). The so formed inflammatory milieu exerts autocrine and paracrine effects responsible for the recruitment of circulating wild-type inflammatory cells to lesion sites, and for the induction and maintenance of the senescent phenotype in both mutated and bystander infiltrating cells. Cytokine-blocking agents interfere with the inflammatory effects downstream BRAF^V600E^ and exert moderate efficacy in the treatment of ECD, but are ultimately of no cure for the disease. Conversely, therapy with the selective BRAF^V600E^ inhibitor vemurafenib might dampen all the pathogenetic mechanisms of ECD.

Collectively taken, these data support a central role of BRAF^V600E^ in the pathogenesis of ECD, and suggest OIS as the possible link between the oncogene mutation and the observed inflammatory activation. As previously described, ECD recapitulates *in vivo* the molecular events associated with OIS. According to this model, ECD is a clonal disease of macrophages bearing the BRAF^V600E^ mutation. The activation of OIS programs in mutated cells results in an increased production of pro-inflammatory cytokines, thus inducing potent local and systemic inflammatory effects. The infiltration of different tissues by macrophages and the inflammatory local and systemic effects observed in ECD likely represent a drawback of OIS (Figure 2). Thus, the paradigm of ECD pathogenesis delineates OIS not only as a protective pathway against overt cancer development, but also as a new mechanism intrinsically responsible for disease development. It is however tempting to speculate that OIS, although responsible for the local and systemic alterations seen in ECD, may prevent ECD cells to overtly proliferate and thus to give origin to a more aggressive and invasive phenotype, in agreement to what observed in LCH (49).

To date, whether ECD is a cancerous process or a disease characterized by recruitment and activation of histiocytes remains a matter of debate. Is ECD a clonal or an inflammatory disease in nature? Most likely, it is both. Indeed, the emerging evidence on BRAF^V600E^ mutation delineates a new conception of ECD, which encompasses all previous pathogenic theories. Moreover, this model not only reconciles the dichotomy between clonal and inflammatory pathogenesis, but also explains why several therapeutic approaches explored so far yielded unsatisfactory results. As BRAF^V600E^ mutation seems to contribute to ECD pathogenesis through OIS, it is tempting to speculate that specific targeting of senescent cells may hold promise as a future treatment for ECD.

Whereas the mere disruption of pathways responsible for senescence-associated replicative arrest could promote cancer development, strategies that eliminate accumulating senescent cell might instead be beneficial, both by dampening tissue inflammation and damage, and by eliminating cells bearing potentially cancerous lesions, thereby reducing cancer risk. The characteristic phenotype, gene expression, and secretion patterns of senescent cells make them a suitable target for *ad hoc* designed antibodies or small molecules (50). Since targeting senescent cells is emerging as a possible therapeutic strategy to delay or prevent several age-related or chronic diseases, future discoveries and developments in this field will hopefully translate into new treatment options for ECD.

## Conflict of Interest Statement

The authors declare that the research was conducted in the absence of any commercial or financial relationships that could be construed as a potential conflict of interest.
